# Elderly and forced displacement in Colombia

**DOI:** 10.25100/cm.v50i2.4009

**Published:** 2019-06-30

**Authors:** Carmen-Lucía Curcio, José Hoover Vanegas, María Cristina Palacio, Jairo Corchuelo Ojeda

**Affiliations:** 1 Universidad de Caldas, Facultad de Ciencias para la Salud. Departamento Clínico, Grupo de Investigación en Gerontología y Geriatría. Manizales, Colombia.; 2 Universidad Autónoma de Manizales, Grupo de investigación Cuerpo-Movimiento. Manizales, Colombia.; 3 Universidad de Caldas, Manizales, Colombia.; 4 Universidad del Valle, Facultad de Salud, Escuela de Odontología. Cali, Colombia.

**Keywords:** Old age, quality of life, armed conflicts, symbolic interactionism, violence, forced displacement, Colombia, Aged, Life change events, refugees, Vejez, calidad de vida, conflicto armado, interacción simbolica, violencia, desplazamiento forzado, eventos en los cambios de vida, refugiados, Colombia

## Abstract

**Objective:**

To describe the experiences of older adults around forced displacement due to the Colombian armed conflict.

**Methods:**

Interpretive-comprehensive study, with a hermeneutical approach; several types of sampling were carried out. The participants were 12 people aged over 60 years, who reported having being displaced and who participated in the SABE Colombia Survey. The data were encoded using the Atlas.ti software. A process of condensation of central analytical, support and emerging categories was made.

**Results:**

The displacement generated by the armed conflict has been decisive in the current life conditions of the participants. They know that they are survivors of someone else's violence; there is dislocation, loss of territory, de-anchoring, lack of protection and insecurity. To the stigma of old age, it is added being displaced and being strangers in a place where they don’t belong. They live the violent uprooting of their lands and the confusion of their identity; they found themselves in a foreign scene where they were the unusual and the strangers; from receiving threats, they passed to be labeled as ‘threatening’. This forced displacement stems from violence, but also from fear, and it marks the trajectory of life for older people who experience a prolonged struggle for survival in often hostile environments, living "permanently" displaced.

**Conclusion:**

When there is displacement, older people are not only shed of their land and their home, but also from their cosmos and their vital referents; in addition, it changes their life trajectory and their place in the world. Interventions should be designed based on specific particular and contextual analyses.

## Resumen

**Objetivo:**

Describir las vivencias y experiencias de adultos mayores en torno al desplazamiento forzado por efectos del conflicto armado.

**Métodos:**

Estudio interpretativo-comprensivo, con un enfoque hermenéutico. Los participantes fueron 12 personas mayores de 60 años que reportaron haber sido desplazadas y que participaron en de la Encuesta SABE Colombia. Los datos se codificaron utilizando el software Atlas.ti. Se hizo un proceso de condensación de categorías analíticas centrales, de soporte y emergentes.

**Resultados:**

El desplazamiento generado por el conflicto armado ha sido decisivo en las actuales condiciones de vida de los participantes. Saben que son sobrevivientes de una violencia ajena, hay desubicación, desterritorializacion, desanclaje, desprotección, inseguridad. Al estigma de la vejez se le suma el ser desplazados y extraños en un lugar donde no tienen lugar. Viven el desarraigo violento de sus tierras, la confusión de su identidad, se encontraron en un escenario ajeno donde ellos eran los raros y los extraños, de recibir amenazas pasaron al señalamiento de ser amenazantes. Este desplazamiento forzado nace de la violencia, pero también del miedo y marca la trayectoria de vida a las personas mayores quienes experimentan una prolongada lucha por la supervivencia en entornos a menudo hostiles, viven desplazados "permanentemente".

**Conclusión:**

Cuando hay desplazamiento, las personas mayores no solo se despojan de su tierra, de su casa, sino de su cosmos, de sus referentes vitales y se modifica su trayectoria de vida y su lugar en el mundo. Las intervenciones deben ser diseñadas basadas en análisis particulares y contextuales específicos.

**Remark**

**Table:** 

1) Why was this study done?
We want to contribute to a better understanding of the experiences of older adults in relation with forced displacement due to the effects of the Colombian armed conflict, which serves as a basis to allow the generation of strategies that contribute to transform the discursive practices that make them invisible, as well as to favor their inclusion and their development, in conditions of dignity and quality, in order to promote active and healthy aging.
**2) What did the researchers do and find?**
This interpretive-comprehensive study, with a hermeneutical approach, show the vulnerability, exclusion, marginalization and inequalities of older adults who have in their history the experience of forced displacement. To the stigma of old age, the burden of destroying its territorial reference and the experience of a strange and hostile habitat is added to their survival. They changed the logic of daily life, they feel poverty, hunger, uprooting. Four words are repeated: fear, save, abandon and survive.
**3) What do these findings mean?**
Displaced older adults, wrapped in their historical-cultural memories, carry their nostalgia for what was taken from them, the only heritage they have to deal with the condition of physical, economic and emotional fragility; they carry with them a series of knowledge and strategies that were key to them in their previous life, which definitely marked the way of being, inhabiting and building a space and a place in the world. The neighborhoods where they arrive become a social-relational space where there converge duels, fear, anguish, hopes, life projects and diverse regional identities; their voices must continue to be heard.

## Introduction

Displaced people are all subjects who had to move out within the national territory due to situations of internal armed conflict, internal disturbances or tensions, widespread violence, massive violations of Human Rights and International Humanitarian Law ^1^ ^-^ ^5^. Displaced people, unlike refugees, while remaining inside their country, remain subject to the legal and institutional regime and the laws of their own State ^2^ ^,^ ^6^.

Forced displacement is configured as an exodus process that involves many changes, transformations and impacts. It means a process of loss of territory, not only in geographical terms but also cultural, political and legal ones ^5^. It generates the violent rupture of family networks, which causes a growing irreversible social de-structuring, and the rupture of cultural referents. This phenomenon affects the integrity of the victims and generates psychological problems, economic losses, damage to social and community networks that affect individual capacity and possibilities ^1^ ^,^ ^2^ ^,^ ^5^ ^,^ ^7^ ^,^ ^8^.

The annual report of the Agency of the United Nations Organization for Refugees ^9^ shows that forced displacement in 2017 reached a record 68.5 million people; that is, on average one person was forced to leave their home every two seconds, being developing countries the most affected. According to the same report, in 2017 Colombia remained the country with the highest population of internally displaced persons, with 7.7 million, with an increase of more than 250,000 since the beginning of the year. Syria appears in second place, despite having decreased from 6.3 million in 2016 to 6.2 million in 2017. In the Democratic Republic of the Congo, the number of displaced people continued to increase from 1.6 million in 2015 to 4.4 million in 2017. Other countries with internal displaced populations exceeding one million reported at the end of 2017 were Yemen (2 million), Sudan (2 million), South Sudan (1.9 million), Afghanistan (1.8 million), Ukraine (1.8 million), Nigeria (1.7 million) and Ethiopia (1.1 million) ^9^. 

Forced displacement in Colombia presents differences in its causes. It brings together the causes of displacement observed in other countries; this multi-causality makes it deeper and more complex ^2^. Up to four types of displacement stand out: the political, by pressure of the guerrillas, paramilitary forces and drug traffickers; and a large part of the displacements are multi-causal. Another difference is the duration time; in Mexico it is medium term; only from the 70s, and especially in the 90s, the insurgent groups generated internal armed displacement. In Honduras the phenomenon is recent; starting in 2009, displacement was identified as a result of widespread violence. In Peru, displacement covered two decades and ended in the 1990s. In Colombia, it has a long-term historical trend of approximately 70 years ^2^ ^,^ ^5^ ^,^ ^10^. Due to the number of people affected in Mexico and Honduras, displacement has been a drop by drop process. In Peru, and especially in Colombia, it has been massive. With regard to coverage, the most affected area in Mexico is the northeast of the country; it initially affected the indigenous population, and now affects the entire population; in Honduras it occurs in urban areas and affects the general population; in Peru, it was the *Altiplano* (highlands) area, preferably the indigenous population; and in Colombia, it has an impact on the entire national territory, and generally affects the peasant population ^11^. Thus, in Colombia, forced displacement has been a prolonged, multi-causal, intense and growing process that affects all population groups ^10^ ^,^ ^12^.

The elderly represent 8.5% of displaced population in Colombia ^13^. According to the Single Victims Registry, forced displacement represents 73.5% of all crimes that affect the elderly in the development of the armed conflict ^14^. According to the SABE Colombia study ^15^, 15.4% reported having been displaced at some time due to violence or armed conflict. The median age of the first displacement event was 50 years. Differences were observed in the occurrence of displacement according to ethnicity, region and socioeconomic status. The regions where displacement was most frequently reported were the Orinoquia/Amazonia (26.6%) and the Atlantic region (21.0%). It was also reported in all strata; but while 23.9% of the population of social strata 1^1^ had been displaced, it corresponds to 10.7% in strata 5 and 6. Among those who reported being displaced, the majority (87.3%) suffered it more than once ^15^.

Older people face differentiated, disproportionate and accentuated risks due to the armed conflict, both at the time of displacement and during their life trajectory ^10^ ^,^ ^16^.

The situation in which displaced older adults find themselves demonstrates a high degree of discrimination and vulnerability that aggravates their conditions of existence and demonstrates the failure of the State to grant the necessary guarantees for the restoration of their rights to life, dignity and equality ^17^. The guideline of differential approach of the Ministry of Health ^4^ ^,^ ^18^ states that older people who have been displaced have a different generational trajectory, lived another story, other conditions and other opportunities, developed other abilities and skills. Their family, social, cultural, symbolic and religious world collides and faces a reality that was neither decided nor chosen by them. The "Stop it now!" Report of the Historical Memory Group concluded that older people who are displaced suffer a deep uprooting, as they have few psychological and physical resources to adapt to their new life. Displacement also means a deterioration in their conditions and quality of life, since they migrate to more vulnerable and marginalized urban areas ^10^. Being displaced is something like being on a threshold, separated from an initial point but not yet established at the arrival point.

Despite the increasing information on forced displacement, both in various regions of the world and in Colombia, information on older adults and their experiences is very scarce. Displacement generates a situation of experience, which implies that it does not mark the victims as a different or specific set of population; it is about people who have suffered a particular event that marked their lives.

In this context, the objective of the present study was to describe the experiences of older adults in relation with forced displacement due to the effects of the Colombian armed conflict in the participants of the SABE Colombia study: old age and quality of life ^19^. With this, we want to contribute to a better understanding of the subject, which serves as a basis to allow the generation of strategies that contribute to transform the discursive practices that make them invisible, as well as to favor their inclusion and their development, in conditions of dignity and quality, in order to promote active and healthy aging.

## Materials and Methods

### Design

Interpretive-comprehensive study, with a hermeneutical approach and analysis from symbolic interactionism, which focused on interactions, the dynamics of social activities among people, the meanings they attribute to events, the natural environments in which they live, and the actions they perform ^20^.

### Participants

There were taken all the older persons who reported having been displaced and who participated in the SABE Colombia Survey (2016) in their qualitative component: old age and quality of life of older people in Colombia ^19^. The original study included 123 people aged 60 years and older; three types of sampling were carried out: geographical for convenience, intentional and sampling of cases of maximum variation.

The inclusion criteria were: to be aged 60 years or older and to accept participation by signing the informed consent, not being institutionalized, and without cognitive impairment; for the latter, it was applied the Abbreviated Mini-mental Test. The maximum score obtainable was 19; the cut-off point to be included in the study was 13. Finally, that they did not present any acute illness or communication disorders (in hearing or language). The selection did not respond to a saturation criterion with inferential claims for displaced population, but to an aspiration to exemplify the situation of displaced elderly people in Colombia.

Displaced people indigenous people who participated in this study belong to the Piapoco, Sikuani and Puinave communities. The Piapoco are an indigenous people who live in the province of Guainía, their economy articulates agriculture with fishing and hunting, and the family organization is based on the authority of the father-in-law. The Sicuani are the most numerous indigenous people of the Colombian Orinoquia; they are traditionally semi-sedentary and inhabit jungle areas; artisanal fishing, horticulture, hunting and gathering of wild fruits constitute traditional sectors of the Sikuani economy. For their part, the Puinaves live in the jungle areas of the province of Guaviare, on the borders with Venezuela and Brazil. The Puinave family is nuclear and patrilineal; they basically dedicate themselves to agriculture, which complements fishing ^21^.

### Information log

A semi-structured interview was used at the interviewee's home, with a previously established guide ^22^ ^,^ ^23^. All interviews were audio-recorded and transcribed verbatim in word format, and reviewed by one of the researchers to ensure validity; the data was anonymized, and identification codes were used to ensure confidentiality. The interviews were conducted between February and August 2015. There were also recorded the socio-demographic information (sex, age, schooling, occupation, socio-economic status, members and co-workers in the home, family formation and social security) and the informed consent provided by the ethical support.

### Analysis of data

Data were encoded using the Atlas.ti version 8 software. A process of condensation of central analytical, support and emerging categories was made. The product was the registration of the information and its transformation into qualitative data required for the understanding and interpretation of the experiences around the displacement of the elderly participants, as well as the interweaving of discovery, coding (organization) and relativization scenarios (debugging and condensation), typical of this type of research. 

These are mobile scenarios, they are not prefixed or determined but neither are they spontaneous or random ^24^; they were configured through interactions between the various participating agents (older people, interviewers and research team) in correspondence with the willingness to seek answers to the central focus of the investigation. To ensure reliability and validity, a triangulation of voices (older people) was made; aggregate triangulation, which consisted of the grouping by conceptual code of systematization and informant subject; interactive triangulation to analyze similarities and differences; and finally, a collective triangulation in which the experience and training of researchers and interviewers were taken into account. In addition, there were documented all epistemological, theoretical and methodological decisions, taken throughout the study, from initial conceptualization to study design, sampling, analysis and reporting, in order to provide transparency and facilitate external evaluation that was carried out by professionals from the Ministry of Health.

The results are presented as a descriptive narrative, since it facilitates generating, organizing and analyzing the empirical evidence about the singular events, and it considers the context to give them meaning in a particular scenario in which the research team and the experiences narrated by the participants interact ^( 25)^, and it allows rescuing the values ​​of subjectivity ^26^ ^,^ ^27^. The narratives allow us to understand what people think, believe and feel about realities such as violence, war, coexistence, their perceptions, emotions, and the way they are perceiving the changes and situations to face in their daily lives. The narratives are presented in four sections: between indignity and survival; the drift of older people; displacement opens space but reduces existence, and between anguish and fear.

## Results

Twelve people reported being displaced by armed conflict, three indigenous and nine peasants; two of them were displaced more than once. The age range was between 61 and 85 years. Nine of the participants came from rural areas of the country; the majority were small farmers, three of them displaced from the Sierra Nevada de Santa Marta and surrounding areas, who currently live in a settlement for displaced people in the municipality of Fundacion, on land donated by the City Hall, in wooden houses without public services. Two of the respondents live in Cali and were displaced from Magüí Payan, a municipality in the center of the province of Nariño. They moved to the Municipality of Tumaco; from that place, they were displaced again. Three of the participants currently live in Bucaramanga; two of them, farmers, come from the Catatumbo region; the third one, a woman housewife, was displaced as a result of the kidnapping of her husband; she comes from the province of Santander. The latter was the owner of a cattle farm in the eastern plains; he currently lives in a shelter in the municipality of Villavicencio.

The displacement generated by the armed conflict has been decisive in the current living conditions of the participants. Everyone has in common that they were born and raised outside the urban world they now inhabit. All were displaced between 2001 and 2011, and all by the presence of the armed conflict in their territories. Four words are repeated in these voices: fear, save, abandon and survive. Because of fear, they left their territory. To save their own lives, and the ones of their families, they left. Leaving behind their houses, lands, jobs and animals was the only option. Survival is the only (thing) remaining for them. 

These stories sharpen the look around the vulnerability, exclusion, marginalization and inequalities of older adults who have in their history the experience of forced displacement. To the stigma of old age, the burden of destroying its territorial reference and the experience of a strange and hostile habitat are added to their survival. They changed the logic of daily life, they feel poverty, hunger, uprooting. The reason for their violent departure was the defense of their family, their children, so that they would not be taken away or made disappear; but in the city, they have other fears and other threats: insecurity, drugs, prostitution, and loss of authority. Health changes its meaning in the city; in the countryside, institutional resources are restricted, but nature compensated for the shortcomings; and for older indigenous people, their daily practices are entangled and confused in the logic of the market, private appropriation and rationality of other groups. They live loneliness with indifferent strangers; violence and displacement took away the certainties of their community ties, and put them before an individualism that isolates them even more. However, they feel they have to survive, from the resignation they bear in their own memories.

### Between indignity and survival

They lived the violent uprooting of their lands, the confusion of their identity as a guarantee of survival; they found themselves in a foreign scene where they were the unusual and the strangers; from receiving threats, they passed to be labeled as ‘threatening’. “In my community, in my village, we didn't feel poor ... we had freedom and independence ... here, there is a lot of insecurity ... people look at us in a weird way ... we have other fears ... yes ...” (..); a scenario in which time to live "... no longer guaranteed the strength to lift (to plow) the earth" (H68). Also, once (they are) displaced, they are (displaced) again: “*It was hard for me, because I did not move out from only one part, I moved out from one part first; and then, I don't even know how many times*” (M66). An abrupt dynamic that quickly changed their world and their lives as a result of interventions by various agents of the armed conflict: state (army and police), para-state (paramilitary forces), anti-State (guerrillas), in addition to common criminals. The death and disappearance of a close relative, the use of direct or veiled threats, even the fear of a latent proximity put them in the option of saving (themselves) or dying: “(…) we had to leave when these people arrived… in order to survive ... so that (our) children would not be taken” (M61). “*I had to leave because that conflict began, that the armed groups attacked the law (that’s how peasants sometimes call the official armed forces); then, one in the middle of the fight, so what we had to do was to leave the farm, because of the fear that they would take us, in order to kill us* (H77-1).

For the indigenous people, it implies a double cultural shock; on the one hand, changing their daily practices, habits and routines by “(…) going to the civilization of the white people” (M66), with customs as strange as the use of money to buy things, facing the sense of the private (property), not eating what nature offers but what was (available) in the grocery stores. On the other hand, a displacement of the ancestral memory that legitimized their symbolic place as older people in the community; their wisdom is useless in the new culture.

But this outcome was also lived by the elderly peasants. It is that the land catches you, it links directly with nature, and their long history in the countryside gave them assurances and made possible to some extent the satisfaction of basic needs: "*In the city, we do know (about) enduring hunger*" (M66); “*Here (in the city), there is no work for us, we are a burden for our children, who welcomed us; although they already have their lives done*” (M66). “*I used to live in a ranchito (a humble, small house), I was able to (catch) fish; the countryside is more bearable; in towns instead, the people are screwed, they need to have (lots of) money, in order to live well*” (H73). 

The abrupt flight was not easy for the elderly "(...) I cry a river (a lot) ..." (H63). Anchoring weighs on them "*Very sad, every little thing that one has gotten in life (by) working; and we had to leave it (everything), because of the violence*" (M61). And in the face of a shortening future, there is resistance to displacement; they have longing, but not the expectation of returning; they are resigned " *They say that there will be a return, we have gone to see, to do something out there, but there are no more forces (we don't have remaining strengths)* ..." (H77-1). However, they are not expressive in the face of this past, they avoid talking about the facts "When it comes to me ... those displacements sometimes my tears get out (I cry), I would not finally like to tell (about it) ... but then, because it is (about) this talk, I will tell you my case” (H63). The guilty (persons) are not point out, (only) silence speaks. They name the "kids" (guerrilla people), the "commanders" (of paramilitary and military forces), but indiscriminately; and justify the violent departure in order to protect themselves, their children and their family. “*We had to leave that (everything) abandoned and come here with what we were wearing, nothing else, in order to save (our) lives*” (H77). "*My father was kidnapped by the guerrillas, and they began to chase my children*" (H67). 

For this reason, forced displacement confuses the elderly, they know why they are leaving, but this does not prevent them from recognizing the perspective of the time they have, they feel the proximity of finitude, the dispossession of their roots, the fracture of their history, and they do not have time or energy to start over, "We were going to sell the ranchito, in order to leave and see if we could revive something, but there were only weeds, mere mountain *(wild vegetation), then we had to come here, waiting for nothing.*" (H77). They only know that they are survivors of a violence external to them, that they did not provoke or were part of it, but that made them to bear the stigma of being displaced and strangers in a place where they have no place, “*I am from the jungle… one always miss (the place) where we belong ... where we left from*” (M66).” 

### The drift of older people

Displacement in human beings, rather than uprooting, rather than forced mobility, is dislocation, (decentralization, loss of territory, de-anchoring) de-protection, insecurity; especially for the elderly, who have traveled through life around a house and a home.

When there is displacement, older people are not only shed their land, their home, but their cosmos, their vital referents. “(…) *But the hard part was moving out from Tumaco towards here, so my house is over there*.” (H74). The image that they have built of themselves, and that has allowed them to differentiate themselves from others, and at the same time be recognized by others, has become unstructured and reconstructed in light of the new realities and social positions that they are obliged to assume.

Thus, the counter-face of trust, security and tranquility is constituted by forced displacement. The elderly have accumulated a large number of experiences, many of which become a mental mapping of the space that constitutes their personal heritage, an effective means of survival. These mind maps are elaborated through the repetition of routes; the space is learned, and it is actively learned from space; and it is well known that the ability to construct spatial images in the short term constitutes a real problem with aging; therefore, changes of address and the uncertainties about the house produce confusion, loss of important elements of spatial reference, often related to emotional memory, derived from the process of buying a house or building it. This aspect becomes vitally important in a situation of displacement, "*... it’s bad, one remains* as trambuleco *(confused), feeling bad, having to abandon what one had ...*"; especially when the displacement is prolonged: "*... we have been here for over 10 years, that for a season (we thought it was going to be for a short period of time) and see, it’s been 10 years and nothing ... (we are) bad, but we have to, because what else we are going to do, see how low the house is, the heat is tremendous, and see the little wood is already rotten, we have it tied with nylon, because winter comes in and all this becomes flooded.*” (H77). However, this situation of loss and displacement also gives way to solidarity “*There are many displaced people who had land (but now) are scrubbed here. I invaded here; (then,) I met a guy who had 7 relatives (but) had no place to live; he was enduring hunger. I told him: take that invasion; there, I leave it to you; I’ll take some leaves and make my cambuche (hovel)*” (H68). 

The house is the reference site; when the elderly are stripped of their home, they are morally misplaced; every human being needs a center of existence, a place of reference, from where to leave, where to arrive, a zero point that nourishes their sense of life. The exit, the displacement, marks a breaking point in social relations, in the way of life, particularly affected by the loss of housing, and with it, the basic conditions of family unity, autonomy, privacy and protection. The impact of this loss of reference elements goes beyond the security of the house, until limits that touch the identity itself “*…I lived there with my children; then, when that happened that day, we flew away, with what each one could grab, (then) each one left towards there, here ... because I did not understand anything, I had not met them; when they caught me, I did not know their name or anything, when they asked me "so... you do not have an identity card, their children", ... "No, I have nothing", is that I missed myself* " (M85).

When the house and the existence get opposed, life prevails by nature: “*I had to leave because that conflict began, that the armed groups attacked the law; then, one in the middle of the fight, so what we had to do was to leave the farm, because of the fear that they would take us, in order to kill us*” (H77); “… Here in Santa Rosa there I lived, my children had them there, the house was not very big but we always lived and that I left behind because they killed a little there, they were taken in a car full of blood, they took healthy people who they were not bad, so I flew away, since I had no man, they had killed me in that town” (M85).

Between the house and the life, between the location and the dislocation, between the security of the home and the insecurity of displacement, life prevails, “*I ran away from them and… the, the welcome they gave me was that the same night that I got there, to Cali, they robbed me; there in ..., (just) inside the city’s bus station, they stole my suitcase, the money that I had, the cell phone, the hat, a pair of cachiri leather boots, the poncho, they left me walking in stockings, one remains like that, see; I also had to go to sleep over there in a shelter for beggars ... (H67). "... that you feel ... if you do not move, you’re going to die ... that is difficult*" (H74).

The house is the zero point that houses the elderly; faced with displacement, this point fades, and the return becomes an increasingly dark hope: “*At the moment when I left displaced because I was hoping to find a job over there; that there, in Cali, I could get a job in some farm or something, even though I did not realize that I was deceiving myself; but there I was, I felt that hope until the moment I left that place*” (H74). Returning is always less likely, the way back has lost the mark of the elderly who left them because of the rush of flight; displacement opens one path to leave, but not another to come back. Displacement ends with the hope of returning.

### Displacement opens the space but reduces the existence

Displacement opens the road, which used to have a limit and a point of arrival, towards an infinite horizon, without limits. Displaced older people travel the land without a place to reach. Any place of arrival becomes a place of departure: "*We were here for a while, and then over there*." (H63). The arrival sites become transit sites, any point is nothing, the world has no borders for displaced older people, “Right now, I am in a shelter intended for the homeless; before [living in the shelter], I had to sleep in the street (…) I have been displaced during four years” (H67). 

In the scenario of the open space that generates the displacement of the elderly, there appears *the other*, the strange thing, the foreigners. This implies a loss of the *habitual*, a de-familiarization, a deterioration of the neighborhood; leaving the own home, leaving the land that houses and protects them implies accessing another space, full of other people, of strangers. The others are not the same, they are other faces, without familiarity, without friends, which makes them uneasy, and (which) alters one's security, especially for a doubly vulnerable person: elderly and displaced adult. Being a neighbor again, making friends again, rebuilding a routine for older people is often more difficult than for young people. Displacement leads older people to a strange world, a world that must be rebuilt, adjusted again, conquering strangers as partners, as neighbors again. Displacement implies to build the world again, to rebuild the home: "*... I no longer have to walk around as I was before, thank God I am collected (living) here, inside these three sheets of zinc*" (H77). 

Therefore, forced displacement marks the trajectory of life for the elderly by subjecting them to a scenario of complex violations, which translates into “*The imposition and obligation to change horizons at this stage of life*” (M66). The violent uprooting of everyday space, the abrupt ruptures of their usual scenarios, the disappearance of their social and family supports, the strangeness of traveling through a territory that in principle receives them with hostility, having to face the imposition of starting over.

They went to the city to survive and restart. But displaced people, indigenous and peasants, given their conditions, can only enter the neighborhoods that are part of the so-called slum belts, they (have to) move from rural areas to urban overcrowding; from neighborhood relationships known for years to relationships with strangers and anonymous people. Coming from traditional communities, they face a modern city, more heterogeneous and complex than a village or small town, places that bring together a large variety of people from different regions, climates and customs, whose common denominator is poverty. They evoke the river, the mountain, the animals, which had become part of their way of life, not only because they had been a source of subsistence, but also the work base that had allowed them to be recognized as independent people and who respond for their families, which had provided them with social and individual identity. In the city, they endure hunger, conflicts, shocks, difficulties, destruction, unknown demands, and reconstructions both individually and collectively. "*In the city we do know (about) enduring hunger" (M66); “Here (in the city), there is no work for us, we are a burden (…)” (M66). “I was able to (catch) fish; the countryside is more bearable; people, instead, are screwed, they need to have (lots of) money, in order to live well*” (H73).

### Between anguish and fear

The awareness of existence in old age is to see how the past is widening, and how the future is shortening. If this is true, it is also true that in the trajectory of older people there is more that has been lived than what remains to be lived; this, seen with the eyes of displacement implies a change of ends, interests, hopes; in other words a change of the meaning of life, when not a complete annihilation. In the scenario of displaced older people, unlike people in other stages of life, feelings and passions emerge with a much stronger intensity, since the time to rebuild the world and its meaning is running out. And while for displaced older people the path opens to infinity, narrowness is more frequent. While the future is reduced, the physical world expands.

Forced displacement of the elderly is revealed between living or dying, security and insecurity, loneliness and company, the friend and the stranger, closeness and remoteness, the infinite horizon or the road and the impossibility of returning, the conservation of the past and the indecipherability of the future, the inside and the outside, narrowness and width, the intimate and the public, the nameable and the unmentionable, the darkness and the light. Each of these pairs of phenomena are present, as feelings of displaced, as an arsenal of events that configure experiences “*What I miss is the way of living there. Tumaco was more peaceful at the beginning, as those groups were gradually taking the cities; because, mainly because ... over there, the people who you saw, you knew them; here, in the settlement, the same native people are becoming evil ... the same persons you know, the ones from the same land, they spend their time doing evil*” (H74). If any phenomenon can be revealed in displaced older people, it is instability; rather than as a spatial or temporal category, it is a mood phenomenon, which does not find regularity in any scenario since the scenario simply does not exist anymore: “…*see, do you know what? It's 2 pm; at 12 am (midnight), we hope not to see any (member) of their family; and the order, boys, is that after 12 am, destroy whatever you find of this family; so we now know it. What do I want to tell you with that? Leave the province of Arauca, you are not a pleasant person for us*” (H64). 

Older people have been displaced by force, but also by fear; fear as the cause of displacement itself and fear of a dark, threatening future. Fear as a consequence of the threat. The certainty of a danger in the near future, leaves no alternative, escaping is the safest, the most reasonable in the absurdity of violence: “*A lot of death, that; then, they almost killed us. At a point in time, when the boy was a baby, they came shooting people and the ranchitos there; from then on, we got very scared*” (M66).

On the other hand, fear emerges as a consequence of the displacement itself, because of the uncertainty of the future, because of what one must face that is still unknown, because of the expectations of what is unfamiliar. Facing a new world opens the door of intimidation, which is a form of fear " *At three in the morning, came a gentleman in a truck; we took the bags, the kids, got into the truck and left, towards Pore, Casanare*" (H67). " *It became very ugly, and one did not even sleep, thinking; then, all the people left, that was left alone ... Then, one also get scared and goes out, because, what (else) can we do?*" (H73). Fear as a cause and as an alternative to an uncertain future predominates in the elderly displaced by the armed conflict, and it permanently accompanies them, as it is evident in the voices provided by the narratives.

The road opens the space of the unknown; returning is no longer possible, “*I would not like to live in the countryside because of those details; if there were no violence, it would be very nice, because everything would return to normality: for the food, the animals, chickens, pigs, everything. But now, if you have a chicken, they take it away, they steal it. Yes, there is no way to live in the countryside*” (H66).

## Discussion

The logic of the armed conflict and forced displacement puts older people facing another suffering of social origin: the swampy terrain of uncertainty and the loss of their identity anchors ^28^. The uprooting caused by violence modifies the life trajectory and dismantles the identity of the elderly. The loss of the house or home means more than losing the home or place of residence, also the domestic environment, the social fabric in which they lived, affections, customs, geography and, above all, the disappearance of their place in the world ^29^.

Displaced people face unfamiliar environments, they must assume new attitudes and activities under difficult emotional conditions, marked by losses and uncertainties. However, this situation is not exclusive to the elderly; Bello ^30^, in a study with displaced families, mentions that being displaced means having lost their place in the world, ceasing to be and being in the place where they had been; it is synonymous with uprooting, anonymity, pain, anger, stubborn presence of memory and effort of forgetting. On the other hand, Ocampo *et al*. ^5^ show that the contents accumulated in historical memory as a group or community, which gave meaning to existence and contributed cognitive and affective elements to define a place for themselves in the world, are transformed as referents of life orientation every day. In this context, people who are victims of forced displacement experience the loss of fundamental elements as individual and social subjects; they are forced to rework their identity each time and to reaffirm what they are, deep within themselves, with their memory and their illusions, beyond immediate survival. 

Although farmers and indigenous people are displaced from rural to urban areas, the impact of this phenomenon is not the same. In Colombia, the definition of rurality has been, typically, the residue or complement of the official definition of urbanization or municipal head ^31^. The census positively discriminates in some cases the Afro-Colombian and the indigenous population, but not the “peasantry.” It is often said that the category "peasant" is ambiguous. Part of the problem is that statistics do not adequately recognize the participants, nor the political phenomena, showing once again the non-valuation of the peasantry by the State ^32^; the same does not happen with the indigenous population.

One of the groups that has been particularly affected by forced displacement and expropriation of their ancestral territories are the indigenous peoples and Afro-Colombians, some of them being on the verge of extinction ^33^. The territory is part of the indigenous universe, and the indigenous is an integral part of the territory; their process is indivisible. The territory is not conceived without the natives, and the indigenous are necessarily bound to their territory; this consideration holds the differentiating concept between indigenous people and peasants; it marks the use of the territory as the fundamental axis of survival of their lives; and not of their model or lifestyle. The territory contains a divine ancestral origin, historically located and identified from its own accounts of origin, room, use and existence. The territory, from its conception, will never be the subject of an economic negotiation on its area or part of its area, it will not be sold; and never, a purchased space will be considered as territory. It gives rise to a wide range of knowledge and daily values ​​that give meaning to its cultural structure ^34^. The mountains, the rivers, the deserts and the jungle are not mere geographical accidents, but resources to which they have historically been attributed meanings and functions of regulation and protection. Their physical destruction, as well as their inappropriate use, represent for many indigenous and peasant communities the devastation of their knowledge and protection systems. 

On the other hand, in Latin America there are more than 800 indigenous peoples that have enormous territorial, demographic and sociocultural diversity, but also have a common denominator: exclusion and material poverty, which affects them more intensely than the rest of the population ^35^. For a large part of the indigenous people, the true old age begins when tasks or activities for the maintenance of the family or for the material reproduction of the community can no longer be carried out. The status and social role may increase as they “get older”, since the older ones are the people who treasure the wisdom and collective memory that must be transmitted to young people, in order to ensure the cultural reproduction of the group or people. Therefore, there is no room for a "negative" interpretation of old age, but for cultural continuity. Also, many of these people are the link between local authorities and the community. In addition, due to the migration of young people and adults, the elderly are those who stay in the countryside and support family life projects through economic strategies ^35^ ^,^ ^36^. Therefore, the separation, destruction or transformation of the territory of an indigenous community is the main determinant of generating ruptures at the social, economic, organizational and psychological level. The fracture of these senses and symbolic values, ​​as a result of displacement, have a greater impact than in other population groups, and the adaptation strategies of these minorities to urban life are more restricted. For example, Western ways of valuing processes, materials, objects and jobs with money have a partial application in their lives. Money has value only in the processes in which it relates to its non-indigenous social environment, and generally in populated centers. They combine the primitive forms of exchange of materials for other materials, work for work, or any of these combinations that apply according to the need of each person, and not for the supply (the law of supply and demand). In a situation of displacement, all this reference becomes fractured. The vulnerability of older indigenous persons increases in cities ^37^ ^-^ ^39^.

When the indigenous elders enter the dynamics of the city, the sense of “old age” that has been configured from the cultural substrates of each town and the biographical experience must begin to re-accommodate symbolically with the social structure of the majority society; it is at that point where, for the majority of indigenous peoples, there is a breakdown and main dislocation about the sense of old age and its role within society ^36^. 

While displacement is primarily a strategy of protection and livelihoods, being displaced is not a guarantee of being safe or achieving a sustainable livelihood ^1^. Studies in Colombia and Nepal have found that land ownership increases the likelihood of displacement, suggesting that people who owned the land could expect greater threats from armed agents, and that the fear of being attacked exceeded the risk of losing the land ^40^ ^,^ ^41^. It has been shown that income and economic privileges are negatively associated with displacement in both Colombia and Nepal, suggesting that lower incomes also imply a lower capacity to adopt defensive measures (for example, protection payments) or to withstand threats to their livelihoods ^40^ ^,^ ^41^, a crucial aspect when leaving the countryside to start a new life in an urban environment; The conditions of poverty and marginalization of displaced older adults increase exponentially.

It is evident that the places to which displaced people arrive are inhabited by the most heterogeneous communities, which also include multiethnic and multiregional elements, whose common reference is poverty; historically experienced in the city by people and communities, but unknown to the newcomers, mostly from rural areas, where their basic needs were met daily. The various food sources provided by the countryside and the wide geographical spaces contrast with the urban environments of current coexistence, places where overcrowding and difficulties to ensure daily food are frequent ^1^ ^,^ ^5^ ^,^ ^7^.

Not all displaced people can achieve good levels of economic integration, and even for those who manage to do it, economic integration does not necessarily equate to economic well-being ^1^. Statistical analyses based on data from Bosnia, Colombia and Sri Lanka suggest that suffering displacement has a significant negative effect on economic well-being ^41^ ^-^ ^43^.

Regarding fear, in almost all places where the Historical Memory Group ^10^ carried out its work, victims referred to fear as the most constant and widespread emotion. The victims, even many years after the events, said that despite the passage of time, fear is still present in their lives. Fear, an effective defensive mechanism, becomes a paralyzing and mortifying emotion that prevents some people from carrying out essential activities to develop their lives, such as leaving their homes, walking through the countryside, and meeting their friends. Lira and Castillo ^44^ talk about chronic fear, a contradictory concept in itself, since fear, like anxiety, are specific responses to an internal or external threat perceived by the subject. For the authors, talking about chronic fear implies that it ceases to be a specific reaction to specific situations, and it is practically transformed into a permanent state in daily life for anyone who feel threatened. In the case of those displaced by the armed conflict, it is shown that there is a continuity of fear that marks the experience of displacement. Fear, an emotion that is experienced individually, is socially constructed and shared culturally ^45^; it results from direct experiences with terror, threats and death before having to flee their homes. This emotion is accompanied by feelings of insecurity and anxiety, associated with the displacement paths, the day of exile and with the challenges and uncertainties of the arrival into an unknown environment, or of the possibility of being forceful displaced again ^46^. According to James ^47^, fear organizes affections, which in a context of displacement and changes of notions of anguish, uncertainty, security and hope, gives collective expression to these experiences. 

Although all the participants in this study spoke of the place of origin with sadness and longing, and they want the return, they do not see it as a real option. Arias et al, in a study on the desire to return of displaced people in Colombia, found that land tenure at the place of origin provides an incentive for returning; vulnerable households, particularly those headed by women and those of ethnic minorities, seek to establish themselves at the reception site, and show less desire for returning; those displaced as a result of a direct attack are less willing to return; economic opportunities at the place of origin encourage returning, while economic opportunities at the reception site decrease the willingness to return; and social networks, as exemplified by membership in peasant organizations and collective ownership of land, increase the desire to return ^7^. In addition, the decision to return to the place of origin is, therefore, partially determined by the causes that lead to displacement, and by the process of displacement itself. This selectivity implies that several factors must be taken into account when assessing the determinants of the desire of displaced population for returning; older adults participating in this study belong to minorities and were subject to direct attacks, which increases vulnerability and fear, and it makes that, although they are in very unfavorable conditions, the return to the place of origin is not seen as a viable option. For those who have been victims of forced displacement, the subjective universe gives account of a place that they do not want to return to, because it is synonymous with pain, fear, insecurity and danger, for having left deep wounds. The physical territory, the mountain and the road became the place of war. The natural heritage, the rivers, the vegetation, which gave a meaning of freedom to the place, to the environment in which his life was developed, became a scene of fear and terror.

The sudden expulsion of older adults from their own territory, as it has been shown in this investigation, provides evidence regarding the situation experienced by rural and indigenous people, as members and participants of society; as victims of a loss of power and control over their lives in the whirlwind of armed violence.

According to Lechner ^48^, the interpretation of the past through experiences determines future trajectories, given that there is a relationship between time, space and memory. Fears of the future are born in the past. However, no literature or references on how to deal with the past have been found in the literature on displacement ^49^.

Displaced older adults, wrapped in their historical-cultural memories, carry their nostalgia for what was taken from them, the only heritage they have to deal with the condition of physical, economic and emotional fragility; they carry with them a series of knowledge and strategies that were key to them in their previous life, which definitely marked the way of being, inhabiting and building a space and a place in the world. The neighborhoods where they arrive become a socio-relational space where there converge duels, fear, anguish, hopes, life projects and diverse regional identities; their voices must continue to be heard.

Memory, according to them, should be done in the middle of the war, in order to stop it, to denounce it, to claim, to transform and build peace. Older adults are those who, in general, resent the forced exit with greater intensity, suffer the experience as a deep uprooting, because they have few physical or cognitive resources that allow adaptation. The deterioration of the quality of life, the changes in climate, food and habits increase the feeling of vulnerability and instability. Without certain and known coordinates, people drift away. Neither the landscapes, nor the customs, nor the sounds, nor the colors, nor the smells are familiar to them. Everything deepens the feeling of estrangement. The dilemma between memory and forgetfulness in a traumatic political situation has specific connotations. Remembering the traumatic may be impossible. But forgetting it can also be it, and the memories can come back violently and relentlessly, breaking over and over again. 

These specificities point to the need that the programming of interventions be designed based on an analysis of particular conditions, rather than tools, and with a significant contribution from displaced persons themselves. Context analysis in any given environment should provide information to emphasize lasting solutions.

This study has several strengths; it gives an account of the careful and critical look with which it is necessary to approach the complexity of forced displacement in Colombia, and the vulnerability of a forgotten minority: elderly peasants and indigenous people. It is a carefully designed and executed qualitative study, which narrates the experiences of forced displacement in a population that is almost never taken into account, despite their triple vulnerability: elderly, displacement and (being) poor. Although the study has been only an approach, it would be important to recognize and reconstruct with new groups of older adults their experience and their process, in order to have an open panorama that allows making public policy decisions and generating pertinent recommendations. As one inhabitant of Trujillo (Valle del Cauca province) mentioned to the historical memory group: “*… If you do not speak, if you do not write and do not tell, you will forget; and gradually, you will get covered by the fear…*” ^10^

## Conclusion 

When there is displacement, older people are not only shed of their land and their home, but also from their cosmos and their vital referents; in addition, it changes their life trajectory and their place in the world. Interventions should be designed based on specific particular and contextual analyses.

## Footnotes

1 Strata: A level of a social hierarchy that is distinguished according to such criteria as educational achievement status
