# Supplementary material for: Elderly and forced displacement in Colombia
Source: Colomb Med (Cali). 2019 Jun 30;50(2):52–66. doi: 10.25100/cm.v50i2.4009 (PMC6774583; doi:10.25100/cm.v50i2.4009)
Supplement: Translation

# Translation: Vejez y deplazamiento forzado en Colombia

**Contribución del estudio**

**Table:** 

1) Por que se hizo este estudio?
Se quiere contribuir a una mejor comprensión de las vivencias y experiencias de los adultos mayores en torno al desplazamiento forzado por efectos del conflicto armado, que sirva como base para permitir la generación de estrategias que aporten a transformar las prácticas discursivas que los invisibilizan, así como a favorecer su inclusión y su desarrollo, en condiciones de dignidad y calidad, para favorecer el envejecimiento activo y saludable
**2) Cuales fueron los resultados mas relevantes?**
Este estudio interpretativo-comprensivo, con enfoque hermenéutico, muestra la vulnerabilidad, la exclusión, la marginación y las desigualdades de las personas adultas mayores que tienen en su historia la experiencia del desplazamiento forzado. Al estigma de la vejez, se le suma la carga de la destrucción de su referente territorial y la vivencia de un hábitat extraño y hostil a su supervivencia. Les cambió la lógica de la vida diaria, sienten la pobreza, el hambre, el desarraigo. Cuatro palabras se repiten:*miedo, salvar, abandonar y sobrevivir*.
**3) Que significan los hallazgos?**
El adulto mayor desplazado envuelto en sus recuerdos histórico-culturales carga con su nostalgia por lo que le fue arrancado, único patrimonio con que cuenta para hacer frente a la condición de fragilidad física, económica y emocional; lleva consigo una serie de saberes y estrategias que le resultaban claves en su vida previa, que marcaban definitivamente la manera de ser, habitar y construir un espacio y un lugar en el mundo. Los barrios a donde llega se convierten en espacios socio relacionales donde convergen duelos, miedo, angustias, esperanzas, proyectos de vida, identidades regionales diversas; sus voces se deben seguir oyendo.

## Introducción 

Desplazado es todo sujeto que se ha movilizado dentro del territorio nacional a causa de situaciones de conflicto armado interno, disturbios o tensiones interiores, violencia generalizada, violaciones masivas a los Derechos Humanos y al Derecho Internacional Humanitario ^1^^-^^5^. Los desplazados, a diferencia de los refugiados, en tanto permanecen en el interior de su país, siguen estando sujetos al régimen legal e institucional y a las leyes de su propio Estado ^2^^,^^6^. 

El desplazamiento forzado se configura como un proceso de éxodo que implica muchos cambios, transformaciones e impactos. Significa un proceso de desterritorialización, no solo en términos geográficos sino también culturales, políticos y jurídicos ^5^. Genera la ruptura violenta de redes familiares, lo que provoca una creciente desestructuración social irreversible y la ruptura de los referentes culturales. Este fenómeno afecta la integridad de las víctimas y genera problemas psicológicos, pérdidas económicas, daño a las redes sociales y comunitarias que afectan la capacidad y posibilidades individuales ^1^^,^^2^^,^^5^^,^^7^^,^^8^

El informe anual de la Agencia de la Organización de Naciones Unidas para los Refugiados ^9^ muestra que el desplazamiento forzado en 2017 alcanzó el récord de 68.5 millones de personas, es decir, en promedio una persona cada dos segundos fue obligada a abandonar su hogar, siendo los países en desarrollo los más afectados. Según el mismo informe, en 2017 Colombia seguía siendo el país con más población de desplazados internos con 7.7 millones, con un aumento de más de 250,000 desde el comienzo del año. En segundo lugar esta Siria pese a haber disminuido de 6.3 millones en 2016 a 6.2 millones en 2017. En la República Democrática del Congo la población de desplazados siguió aumentando de 1.6 millones en 2015 hasta alcanzar 4.4 millones en 2017. Otros países con poblaciones de desplazados internos superiores a un millón comunicadas al final de 2017 fueron Yemen (2 millones), Sudán (2 millones), Sudán del Sur (1.9 millones), Afganistán (1.8 millones), Ucrania (1.8 millones), Nigeria (1.7 millones) y Etiopía (1.1 millones) ^9^.

El desplazamiento forzado en Colombia, presenta diferencias en sus causas, en él confluyen las causas generadoras del desplazamiento observadas en otros países, esta multicausalidad lo hace más profundo y complejo ^2^, se destacan hasta cuatro tipos de desplazamiento: el político, por presión de la guerrilla, paramilitar y por el narcotráfico, y gran parte de los desplazamientos son multicasuales. Otra diferencia es el tiempo de duración; en México es de mediano plazo, solamente a partir de los Años 70, y especialmente en la década de los 90, los grupos insurgentes generaron desplazamiento armado interno. En Honduras el fenómeno es reciente, a partir del año 2009 se identificó el desplazamiento como resultado de la violencia generalizada. En Perú el desplazamiento cubrió dos décadas y concluyó en los años 90. En Colombia tiene una tendencia histórica de largo plazo de aproximadamente 70 años ^2^^,^^5^^,^^10^. Por el número de personas afectadas, en México y Honduras el desplazamiento ha sido un proceso gota a gota. En Perú y especialmente en Colombia ha sido de carácter masivo. Con respecto a la cobertura, en México la zona más afectada es el noreste del país, afectó inicialmente a la población indígena y ahora afecta a toda la población; en Honduras se presenta en áreas urbanas y afecta la población en general; en Perú fue la zona del Altiplano, preferentemente la población indígena y en Colombia tiene impacto en todo el territorio nacional y afecta generalmente a la población campesina ^11^. Asi, en Colombia el desplazamiento forzado ha sido un proceso prolongado, multicausal, intenso y creciente, que afecta a todos los grupos poblacionales ^10^^,^^12^.

Las personas mayores representan el 8.5% de la población desplazada en Colombia ^13^(13). Según el registro Único de Victimas, el desplazamiento forzado representa el 73.5% de todos los delitos que afectan a las personas mayores en el desarrollo del conflicto armado ^14^. Según el estudio SABE Colombia ^15^, 15.4% reportó haber sido desplazado alguna vez por la violencia o el conflicto armado. La mediana de edad del primer evento de desplazamiento fue de 50 años. Se observaron diferencias en la ocurrencia de desplazamiento según etnia, región y estrato socioeconómico. Las regiones en donde se reportó con mayor frecuencia el desplazamiento fueron la Orinoquia/Amazonia (26.6%) y la región Atlántica (21.0%). Además fue reportado en todos los estratos, pero mientras el 23.9% de la población de estrato 1 había sido desplazada, en los estratos 5 y 6 corresponde al 10.7%. Entre quienes reportaron haber sido desplazados, la mayoría (87.3%) lo sufrió más de una vez ^15^. 

Las personas mayores enfrentan riesgos diferenciados, desproporcionados y acentuados por causa del conflicto armado, tanto en el momento del desplazamiento como en su trayectoria de vida ^10^^,^^16^.

La situación en la que se encuentran los adultos mayores desplazados, evidencia un alto grado de discriminación y vulnerabilidad que agrava sus condiciones de existencia y demuestran el incumplimiento por parte del Estado del otorgamiento de las garantías necesarias para el restablecimiento de sus derechos a la vida, a la dignidad y a la igualdad ^17^. La directriz del enfoque diferencial del Ministerio de Salud ^4^^,^^18^ plantea que las personas mayores que se han desplazado tienen una trayectoria generacional diferente, vivieron otra historia, otras condiciones y otras oportunidades, desarrollaron otras capacidades y habilidades. Su mundo familiar, social, cultural, simbólico y religioso choca y se enfrenta a una realidad que no fue decidida, ni elegida por ellos. El informe “¡Basta ya!” del Grupo de Memoria Histórica concluyó que las personas mayores que se desplazan sufren un profundo desarraigo, pues cuentan con pocos recursos psicológicos y físicos para adaptarse a su nueva vida. El desplazamiento significa, además, un deterioro en sus condiciones y en su calidad de vida, ya que migran a zonas urbanas más vulnerables y marginadas ^10^. Ser desplazado es algo así como estar en el umbral, separado de un punto inicial y sin establecerse aún en el punto de llegada. 

A pesar de la creciente información sobre el desplazamiento forzado, tanto en diversas regiones del mundo como en Colombia, la información sobre los adultos mayores y sus experiencias es muy escasa. El fenómeno de desplazamiento genera una situación de vivencia, lo que implica que el fenómeno no marca a las víctimas como un conjunto diferente o específico de población, se trata de personas que han sufrido un hecho determinado que marcó sus vidas.

En este contexto, el objetivo del presente estudio fue describir las vivencias y experiencias de los adultos mayores en torno al desplazamiento forzado por efectos del conflicto armado en los participantes en el estudio SABE Colombia: vejez y calidad de vida ^19^. Con ello, se quiere contribuir a una mejor comprensión del tema, que sirva como base para permitir la generación de estrategias que aporten a transformar las prácticas discursivas que los invisibilizan, así como a favorecer su inclusión y su desarrollo, en condiciones de dignidad y calidad, para favorecer el envejecimiento activo y saludable. 

## Materiales y Métodos 

### Diseño

estudio interpretativo-comprensivo, con enfoque hermenéutico y analisis desde el interaccionismo simbólico, el cual se enfocó en las interacciones, la dinámica de las actividades sociales entre las personas, los significados que ellas atribuyen a los eventos, los ambientes naturales en que viven y las acciones que desempeñan ^20^.

### Participantes

Se tomaron todas las personas mayores que reportaron haber sido desplazadas y que participaron en de la Encuesta SABE Colombia (2016) en su componente cualitativo: vejez y calidad de vida de las personas mayores en Colombia ^19^. El estudio original incluyó a 123 personas de 60 años y más y se llevaron a cabo tres tipos de muestreo: geográfico por conveniencia, intencional y muestreo de casos de variación máxima. 

Los criterios de inclusión fueron: tener 60 años o más y aceptar la participación mediante la firma del consentimiento informado, no estar institucionalizado y sin deterioro cognoscitivo, para esto se aplicó el Minimental Test Abreviado, el puntaje máximo obtenible fue 19 y el punto de corte fue 13 para ser incluido en el estudio. Finalmente, que no tuviera una enfermedad aguda, ni trastornos de la comunicación (audición y lenguaje). La selección no responde a un criterio de saturación con pretensiones inferenciales para la población desplazada, sino a una aspiración de ejemplificar la situación del adulto mayor desplazado en Colombia. 

Los indígenas desplazados participantes en este estudio pertenecen a las comunidades Piapoco, Sikuani y Puinave. Los Piapoco son un pueblo indígena que habita en el departamento del Guainía, su economía articula la agricultura con la pesca y la caza, y la organización familiar se funda en la autoridad del suegro. Los Sicuani son el pueblo indígena más numeroso de la Orinoquia colombiana, tradicionalmente son semi-sedentarios y habitan zonas selváticas, la pesca artesanal, la horticultura, la caza y la recolección de frutos silvestres constituyen sectores tradicionales de la economía de los Sikuani. Por su parte los Puinave habitan en el zonas selváticas del departamento del Guaviare, en las fronteras con Venezuela y Brasil; la familia Puinave es nuclear y patrilineal y de dedican basicamente a la agricultura que complementan con la pesca ^21^. 

### Registro de información

Se utilizó la entrevista semiestructurada en el domicilio del entrevistado, con una guía previamente establecida ^22^^,^^23^. Todas las entrevistas fueron audiograbadas y transcritas textualmente en formato word y revisadas por uno de los investigadores para asegurar la validez; los datos fueron anonimizados y se utilizaron códigos de identificación para asegurar la confidencialidad. Las entrevistas se realizaron entre febrero y agosto del 2015. También se registró la información socio demográfica (sexo, edad, escolaridad, ocupación, estrato socioeconómico, integrantes y convivientes en el hogar, conformación familiar y seguridad social) y el consentimiento informado que brindó el soporte ético. 

### Análisis de datos

Los datos se codificaron utilizando el software Atlas.ti versión 8. Se hizo un proceso de condensación de categorías analíticas centrales, de soporte y emergentes. El registro de la información y su transformación en dato cualitativo requerido para la comprensión e interpretación de las experiencias en torno al desplazamiento de las personas mayores participantes, fue el producto y el entrelazamiento de escenarios de descubrimiento, codificación (organización) y relativización (depuración y condensación), propios de este tipo de investigación. Estos escenarios son móviles, no están prefijados ni determinados pero tampoco son espontáneos ni al azar ^24^; se configuraron a través de las interacciones entre los diversos agentes participantes (personas mayores, entrevistadores y equipo de investigación) en correspondencia con la disposición de buscar respuestas al foco central de la investigación. Para asegurar la confiabilidad y validez se hizo una triangulación de voces (personas mayores); triangulación por agregados que consistió en el agrupamiento por código conceptual de sistematización y sujeto informante, triangulación interactiva para analizar similitudes y diferencias y finalmente, una triangulación colectiva en la cual se tomaron en cuenta la experiencia y formación de los investigadores y de los entrevistadores. Además, se documentaron todas las decisiones epistemológicas, teóricas y metodológicas, tomadas a lo largo del estudio, desde la conceptualización inicial hasta el diseño del estudio, el muestreo, el análisis y la presentación de informes, para proporcionar transparencia y facilitar la evaluación externa que fue llevada a cabo por profesionales del Ministerio de Salud.

Los resultados se presentan como una narrativa descriptiva puesto que facilita generar, organizar y analizar la evidencia empírica sobre los eventos singulares y considera el contexto para darles sentido en un escenario particular en la cual interactúan el equipo de investigación y las experiencias narradas por los participantes ^25^ y permite rescatar los valores de la subjetividad ^26^^,^^27^. Las narrativas permiten comprender lo que las personas piensan, creen y sienten sobre realidades como la violencia, la guerra, la convivencia, sus percepciones, emociones y la manera en que están percibiendo los cambios y situaciones a afrontar en su vida cotidiana. Las narrativas se presentan en cuatro apartados: entre la indignidad y la sobrevivencia; las personas mayores a la deriva; el desplazamiento abre el espacio pero reduce la existencia, y entre la angustia y el miedo. 

## Resultados 

Doce personas reportaron haber sido desplazadas por conflicto armado, tres indigenas y nueve campesinos, dos de ellos fueron desplazados más de una vez. El rango de edad estuvo entre 61 y 85 años. Nueve de los participantes provienen de áreas rurales del país, la mayoría eran pequeños agricultores, tres de ellos desplazados de la Sierra nevada de Santa Marta y zonas aledañas y actualmente habitan en un asentamiento para desplazados en el Municipio de Fundación, en un terreno donado por la Alcaldía, en casas de madera sin servicios públicos. Dos de los entrevistados viven en Cali y son desplazados de Magüí Payan, municipio del centro del departamento de Nariño, se trasladaron a Tumaco y de allí fueron desplazados nuevamente. Tres de los participantes actualmente viven en Bucaramanga, dos de ellos, agricultores, provienen de la región del Catatumbo y la tercera, una mujer ama de casa, fue desplazada a consecuencia del secuestro de su esposo y proviene del departamento de Santander. El último era propietario de un finca ganadera en los llanos orientales y actualmente vive en un refugio en Villavicencio. 

El desplazamiento generado por el conflicto armado ha sido decisivo en las actuales condiciones de vida de los participantes. Todos tienen en común que nacieron y crecieron por fuera del mundo urbano que ahora habitan. Todos se desplazaron entre 2001 y 2011 y todos por la presencia del conflicto armado en sus territorios. Cuatro palabras se repiten en estas voces: *miedo, salvar, abandonar y sobrevivir*. Por miedo abandonaron su territorio. Para salvar su vida y la de su familia se fueron. Abandonar la casa, la tierra, el trabajo, los animales fue la única opción. Sobrevivir es lo que les queda. 

Las narraciones agudizan la mirada en torno a la vulnerabilidad, la exclusión, la marginación y las desigualdades de las personas adultas mayores que tienen en su historia la experiencia del desplazamiento forzado. Al estigma de la vejez, se le suma la carga de la destrucción de su referente territorial y la vivencia de un hábitat extraño y hostil a su supervivencia. Les cambió la lógica de la vida diaria, sienten la pobreza, el hambre, el desarraigo. La razón de su salida violenta fue la defensa de su familia, de sus hijos, para que no se los llevaran o los desaparecieran, pero en la ciudad tienen otros miedos y otras amenazas: la inseguridad, las drogas, la prostitución, la pérdida de autoridad. La salud cambia de sentido en la ciudad; en el campo los recursos institucionales son restringidos pero la naturaleza compensaba las carencias; y para las personas mayores indígenas, sus prácticas cotidianas se enredan y confunden en las lógicas del mercado, la apropiación privada y la racionalidad de otros grupos. Viven una soledad con extraños indiferentes, la violencia y el desplazamiento les arrebataron las certezas de sus vínculos comunitarios y los puso ante un individualismo que los aísla aún mucho más. Sin embargo, sienten que tienen que sobrevivir, desde la resignación que soportan en sus propios recuerdos. 

### Entre la indignidad y la sobrevivencia

Vivieron el desarraigo violento de sus tierras, la confusión de su identidad como garantía de sobrevivencia, se encontraron en un escenario ajeno donde ellos eran los raros y los extraños, de recibir amenazas pasaron al señalamiento de ser amenazantes “*En mi comunidad, en mi vereda, no nos sentíamos pobres… teníamos libertad e independencia… …aquí hay mucha inseguridad….la gente nos mira raro…tenemos otros miedos…si…” (..)*; escenario en el cual el tiempo por vivir “… *ya no les garantizaba la fuerza para levantar la tierra*” (H68). Además, un vez desplazados, vuelven a serlo: “*A mí se me hizo duro porque yo no me desplacé de solamente de una parte, yo me desplacé de una parte primero, y después ya ni se en cuantas veces”*(M66)*.* Una dinámica abrupta que les cambió rápidamente su mundo y su vida a consecuencia de intervenciones de diversos agentes del conflicto armado: estatales (ejército y policía), para estatales (paramilitares), contra estatales (guerrillas), además de la delincuencia común. La muerte y la desaparición de algún familiar cercano, el uso de la amenaza directa o velada hasta el miedo por una proximidad latente los puso en la alternativa de salvarse o morir: “*(…) nos tocó salir cuando llegó esa gente...para sobrevivir...para que no se llevaran los hijos*” (M61). “*Me tocó salirme porque empezaron el conflicto ese, de que se encontraron los grupos armados con la ley, entonces uno al medio del pleito, lo que le tocaba hacer es venirse y abandonar la finca porque que miedo que lo cojan a uno a matarl*o (H77-1).

Para los indígenas implica un doble choque cultural, por una parte, cambiar sus prácticas, hábitos y rutinas diarias al “*(…) ir a la civilización de los blancos*” (M66), con costumbres tan raras como el uso del dinero para comprar las cosas, enfrentarse al sentido de lo privado, no comer lo que la naturaleza ofrece sino lo que se encontraba en el mercado, y por otra, un desplazamiento de la memoria ancestral que legitimaba su lugar simbólico como personas mayores en la comunidad, su sabiduría es inútil en la nueva cultura.

Pero este desenclave también lo vivieron las personas mayores campesinas. Es que la tierra amarra, vincula directamente con la naturaleza y su larga historia en el campo les daba seguridades y posibilitaba en alguna medida la satisfacción de necesidades básicas: “*En la ciudad si sabemos que es aguantar hambre”* (M66); “*Aquí* (en la ciudad) *No hay trabajo para nosotros, somos una carga para los hijos que nos acogieron, aunque ellos ya tienen su vida hecha*” (M66). “*Yo vivía en un ranchito, pescaba; el campo es más llevadero, en cambio el pueblo es jodido, tiene que tener uno plata para vivir bien”* (H73).

La huida abrupta no fue fácil para las personas mayores “*(…) yo me lloro todito…”(H63).* Les pesa el anclaje *“Muy triste, toda cosita que uno ha conseguido en la vida trabajando y uno deja tirado en las violencias”* (M61) y ante un futuro que se acorta hay resistencia al desplazamiento, tienen añoranza pero no la expectativa de volver, estan resignados “*Dicen que va haber un retorno, hemos ido a ver, hacer por ahí algo, pero ya no hay fuerzas...”*(H77-1). Sin embargo, no son expresivos frente a este pasado, evaden hablar de los hechos “*Cuando me toca… de esos desplazamientos a veces se me salen las lágrimas, quisiera pues a lo último no contar… pero pues ya porque se trata de esta charla yo le contaré mi c*aso*”* (H63), no señalan culpables, el silencio habla. Nombran a los “*muchachos”* (guerrilleros), a los “*comandantes”* (paramilitares y militares) pero de manera indiscriminada y justifican la salida violenta para protegerse, a sus hijos y a su familia. “*Tocó dejar eso abandonado y venirnos para acá con lo que teníamos puesto, no más para salvar la vida”* (H77). “*A mi papá lo secuestró la guerrilla y ellos comenzaron a andar detrás de mis hijos”* (H67). 

Por esto, a las personas mayores el desplazamiento forzado los confunde, saben porque se van pero esto no les impide reconocer la perspectiva del tiempo que tienen, sienten la proximidad de la finitud, el despojo de sus raíces, la fractura de su historia y no tienen tiempo ni energía para volver a comenzar, “*Ya con el tiempo íbamos a vender el ranchito para irnos, a ver si podíamos revivir algo, pero era puro rastrojo, mera montaña, entonces nos tocó venirnos para aquí, esperando nada”*. (H77). Solamente saben que son sobrevivientes de una violencia ajena a ellos, que no provocaron ni participaron, pero que los puso a cargar el estigma de ser desplazados y extraños en un lugar donde no tienen lugar, “*Yo soy de la selva… siempre a uno le falta donde uno es... donde uno salió*” (M66)”.

### Las personas mayores a la deriva 

El desplazamiento en los seres humanos, más que desarraigo, más que movilidad obligada, es desubicación, (descentración, desterritorializacion, desanclaje) desprotección, inseguridad, especialmente para las personas mayores que han transitado por la vida alrededor de una casa y un hogar. 

Cuando hay desplazamiento, las personas mayores no solo se despojan de su tierra, de su casa, sino de su cosmos, de sus referentes vitales. “*(…) pero lo duro fue el desplazamiento de Tumaco hacia aquí, entonces mi casa está allá*.” (H74). La imagen que han construido de sí mismas y que les ha permitido diferenciarse de otras y al mismo tiempo ser reconocidas por otros, es desestructurada y reconstruida a la luz de las nuevas realidades y posiciones sociales que están obligadas a asumir. 

Asi, la contra-cara de la confianza, la seguridad y la tranquilidad, la constituye el desplazamiento forzado. Los personas mayores han acumulado un gran número de experiencias, muchas de las cuales devienen en una cartografía mental del espacio que se constituye en su patrimonio personal, medio efectivo para la supervivencia. Estos mapas mentales se elaboran a través de la reiteración de recorridos, se aprende el espacio y del espacio de manera activa, y es bien conocido que la capacidad de construir imágenes espaciales a corto plazo constituye un verdadero problema con el envejecimiento; por ello los cambios de domicilio, las incertidumbres acerca de la casa, producen desconcierto, pérdida de elementos importantes de referencia espacial, muchas veces relacionados con la memoria afectiva, derivada del proceso de comprar la casa o construirla. Este aspecto cobra vital importancia ante la situación de desplazamiento, “…*mal, queda uno como trambuleco, como mal, tener que abandonar lo que tenía uno…*” más cuando el desplazamiento se prolonga “…*estamos 10 años y más de estar aquí, que por una temporada y vea, van 10 años y nada… (estamos) mal, pero toca porque qué más vamos a hacer, vea esa casa tan bajitica hace un calor tremendo y vea la maderita ya está podrida, la tenemos amarrada con nailon, porque se entra el invierno y se inunda todo esto*.” (H77). Sin embargo, esta situación de pérdida y desplazamiento también da paso a la solidaridad “*Hay muchos desplazados que tuvieron tierra y están fregados por acá, yo hice una invasión aquí, me encontré con un tipo que tenía 7 familiares y no tenía donde vivir, estaba aguantando hambre, le dije: coja esa invasión, ahí se la dejo, yo paro un poco de hojas y hago mi cambuche*” (H68).

La casa es el sitio de referencia, cuando se despoja a las personas mayores de su casa se desubican moralmente, todo ser humano necesita un centro de existencia, necesita un lugar de referencia, de donde salir, a dónde llegar, un punto cero que nutra su sentido de vida. La salida, el desplazamiento, marca un punto de quiebre en las relaciones sociales, en el modo de vida, afectado muy especialmente por la pérdida de la vivienda y con ésta las condiciones básicas de unidad familiar, autonomía, privacidad, protección. El impacto de esta pérdida de elementos de referencia, va más allá de la seguridad de la casa hasta límites que tocan la identidad misma “… *yo vivía ahí con mis hijos, después ya cuando pasó eso, ese día nos volamos nosotros, cada quien con lo que pudo cogió para allá, para acá… porque yo no entendía nada, yo no los conocía, cuando me llevaron no sabía ni el nombre de ellos ni nada, cuando me preguntaban “aja y tú no tienes cedula, de tus hijos”, …“no, yo no tengo nada”, es que yo me perdí yo*” (M85). 

Cuando se contraponen la casa y la existencia, por naturaleza prevalece la vida: “*Me tocó salirme por que empezaron el conflicto ese, de que se encontraron los grupos armados con la ley, entonces uno al medio del pleito, lo que le tocaba hacer es venirse y abandonar la finca porque qué miedo que lo cojan a uno a matarlo*”(H77); “…*aquí en Santa Rosa ahí viví yo, mis hijos los tuve ahí, la casita no era muy grande pero siempre vivíamos y eso lo dejé yo abandonado porque mataron un poco ahí, los llevaban en un carro lleno de sangre, cogían gente sana que no eran malas, entonces yo me vine volando, como no tenía ya hombre, me lo habían matado en ese pueblo”* (M85). 

Entre la casa y la vida, entre la ubicación y la desubicación, entre la seguridad del hogar y la inseguridad del desplazamiento, prevalece la vida, “*Salí corriendo de huida de ellos y … Ja, la bienvenida que me dieron fue que la misma noche que llegué allá a Cali, me atracaron ahí en el…, adentro en el puro terminal, me robaron la maleta, me quitaron la plata que llevaba, el celular, el sombrero, unas botas de cuero cachiri, el poncho, me dejaron andando en medias, queda uno así, vea; también me tocó ir a dormir por allá en un albergue de puros indigentes…* (H67). “…*que usted sienta… si no se desplaza se va a morir... eso es difícil”* (H74).

La casa es el punto cero que alberga a las personas mayores, ante el desplazamiento este punto se desvanece, y el retorno se convierte en una esperanza cada vez más oscura: “*En el momento cuando yo salí desplazado pues yo abrigaba la esperanza de que, de por ahí en un trabajo llegaba, por allá en Cali podía tener trabajo en alguna hacienda o alguna vaina, aunque yo mismo pues no me daba cuenta que me estaba engañando pero ahí estaba, yo sentía esa esperanza hasta el momento que salí de all*á” (H74). Volver siempre es menos probable, el camino de regreso perdió la huella de las personas mayores que las dejaron por la premura de la huida; el desplazamiento abre un camino de ida y no de regreso. El desplazamiento acaba con la esperanza del volver. 

### El desplazamiento abre el espacio pero reduce la existencia

El desplazamiento abre el camino, que antes tenía un límite y un punto de llegada, hacia un horizonte infinito y sin límites. Las personas mayores desplazadas transitan la tierra sin un lugar a dónde llegar. Cualquier lugar de llegada se convierte en lugar de partida: “*Estábamos un tiempo aquí, y luego allá.”* (H63). Los sitios de llegada se convierten en sitios de paso, cualquier punto es una nada, el mundo no tiene fronteras para las personas mayores desplazados, “*En este momento estoy en un albergue que es para habitantes de calle*, *antes [*de vivir en el albergue*] me tocaba dormir en la calle (…) Hace cuatro años estoy desplazao*” (H67).

En el escenario del espacio abierto que genera el desplazamiento de los personas mayores, aparece *lo otro*, lo extraño, los extranjeros. Esto implica una des-habitualidad, una des-familiarización, un deterioro de la vecindad, salir de la morada de la casa, salir de la tierra que los alberga y los protege implica acceder a otro espacio, lleno de otra gente, de extraños. Los otros no son los mismos, son otros rostros, sin familiaridad, sin amigos, lo que intranquiliza y altera la propia seguridad y más para una persona doblemente vulnerable: adulta mayor y desplazada. Volver a ser vecino, volver a hacer amistades, volver a construir una rutina para las personas mayores suele ser mas difícil que para los jóvenes. El desplazamiento conduce a las personas mayores a un mundo extraño, un mundo que hay que volver a construir, volver a ajustar, volver a conquistar a los extraños como compañeros, como vecinos. El desplazamiento implica volver a hacer el mundo, volver a construir la morada *“… ya no tengo que andar por ahí como andaba antes, gracias a Dios estoy recogido aquí, en estas tres hojas de zinc”*(H77).

Por lo anterior, el desplazamiento forzado marca la trayectoria de vida a las personas mayores al someterlos a un escenario de complejas vulneraciones, que se traduce en “*La imposición y obligación de cambiar de horizontes a estas alturas de la vida*” (M66). Al desarraigo violento del espacio cotidiano, las rupturas abruptas de sus escenarios habituales, la desaparición de sus soportes sociales y familiares, al extrañamiento de transitar por un territorio que en principio los recibe con hostilidad tienen que enfrentarse a la imposición de volver a comenzar de cero.

Se fueron a la ciudad para sobrevivir y recomenzar. Pero los desplazados, indígenas y campesinos, dadas su condiciones, solo pueden ingresar a los barrios que hacen parte de los llamados cinturones de misera, pasan de zonas rurales a hacinamiento urbanos, de relaciones de vecinos conocidos por años a relaciones con habitantes extraños y anónimos. Provenientes de comunidades tradicionales, se enfrentan a una ciudad moderna, más heterogénea y compleja que la vereda o pueblo, lugares que reúnen en un pequeño terreno a una gran variedad de personas de distintas regiones, climas, costumbres, cuyo común denominador es la pobreza. Evocan el río, la montaña, los animales, los cuales han hecho parte de su forma de vida, no solo por haber sido fuente de subsistencia, sino la base de trabajo que les ha permitido ser reconocidas como personas independientes y que responden por su familia, les ha proporcionado identidad social e individual. En la ciudad enfrentan hambre, conflictos, choques, dificultades, destrucciones, exigencias desconocidas y reconstrucciones tanto en el plano individual como colectivo “*En la ciudad si sabemos que es aguantar hambre”* (M66); “*Aquí* (en la ciudad) *No hay trabajo para nosotros, somos una carga (…)*” (M66). “*Yo vivía en un ranchito, pescaba; el campo es más llevadero, en cambio el pueblo es jodido, tiene que tener uno plata para vivir bien”* (H73).

### Entre la angustia y el miedo

La conciencia de la existencia en la vejez, es ver cómo se va ensanchando el pasado y cómo se va acortando el futuro. Si esto es cierto, también lo es que en la trayectoria de las personas mayores es más lo que se ha vivido que lo que falta por vivir, esto visto con ojos de desplazamiento implica un cambio de fines, de intereses, de esperanzas; en otras palabras un cambio de sentido de la vida, cuando no la aniquilación completa. En el escenario de las personas mayores desplazadas, a diferencia de las personas en otras etapas de la vida, emergen sentimientos y pasiones con una intensidad mucho más fuerte ya que el tiempo para volver a construir el mundo y su sentido se agota. Y si bien para las personas mayores desplazadas el camino se abre al infinito, la estrechez, la angostura es más frecuente. Mientras el futuro se reduce, el mundo físico se amplía.

El desplazamiento forzado de las personas mayores se revela entre vivir o morir, la seguridad y la inseguridad, la soledad y la compañía, el amigo y el extraño, la cercanía y la lejanía, el horizonte infinito o el camino y la imposibilidad de la vuelta atrás, la conservación del pasado y la indescifrabilidad del futuro, el adentro y el afuera, estrechez y anchura, lo íntimo y lo público, lo nombrable y lo innombrable, la oscuridad y la luz. Cada una de estas duplas de fenómenos se hacen presentes, como sentimientos de desplazado, como arsenal de eventos que configuran vivencias y experiencias “*Lo que extraño es la forma de vivir allá. Tumaco había más tranquilidad al principio, como esos grupos fueron tomando poco a poco las ciudades, porque principalmente pues… allá la gente que uno ve los conocía y aquí en el asentamiento la misma gente nativa gente se está volviendo mala... el mismo que lo conoce a uno de la misma tierra, se la pasa por ahí haciendo maldad”* (H74). Si algún fenómeno se puede revelar en las personas mayores desplazados es la inestabilidad, más que como una categoría espacial o temporal es un fenómeno anímico, que no encuentra regularidad en ningún escenario ya que sencillamente el escenario ya no existe: “… *vea ¿sabe qué? son las 2 de tarde, a las 12 de la noche esperamos no ver a ninguno de su familia y la orden muchachos es que después de las 12 de la noche de hoy, lo que encuentren de esta familia, arrástrenlo, así que ya sabemos, ¿qué le quiero decir con eso? desocupe el departamento de Arauca, usted no es persona grata para nosotros*” (H64). 

Las personas mayores han sido desplazadas por la fuerza, pero también por el miedo; el miedo como provocador del desplazamiento mismo y el miedo ante un futuro oscuro, amenazante. El miedo como consecuencia de la amenaza. La certeza de un peligro en el futuro cercano, no deja alternativa, la huida es lo más seguro, lo más razonable en lo absurdo de la violencia: “*Mucha muerte eso, entonces, casi nos matan una vez cuando la época que tenía el muchacho bebé, vienen tirotiando la gente allí y los ranchitos, nosotros de ahí para allá cogimos fue miedo*” (M66). 

Por otra parte, el miedo emerge como consecuencia del desplazamiento mismo, por la incertidumbre del futuro, por aquello a lo que debe enfrentarse que aún le es desconocido, por las expectativas de aquello que no le es familiar. El enfrentarse a un nuevo mundo abre la puerta de la intimidación, que es una forma del miedo “*A las tres de la mañana llegó el señor del campero, montamos las maletas, los pelaos, me monté yo y vamos para Pore, Casanare*” (H67). “*Se puso muy feo y uno ni dormía pensando, entonces toda la gente se fue, eso quedó solo*… *Entonces a uno le da miedo y sale uno también, porque uno qué hace*” (H73). El miedo como causante y como alternativa ante un futuro incierto predomina en las personas mayores desplazadas por el conflicto armado y los acompaña permanentemente, como se se hace evidente en las voces que brindan las narraciones. 

El camino abre el espacio de lo desconocido, volver ya no es posible, *“No anhelaría vivir en el campo por esos detalles, si no hubiera violencia sería muy lindo, porque volvería todo a la normalidad: por la comida, los animalitos, las gallinas, los cerdos, todo. Pero ahora si una gallina tiene, se la quitan, se la roban. Sí, no hay forma de vivir en el campo”* (H66). 

## Discusión 

La lógica del conflicto armado y el desplazamiento forzado pone a las personas mayores ante otro sufrimiento de origen social: el terreno pantanoso de la incertidumbre y la pérdida de sus anclajes identitarios ^28^. El desarraigo ocasionado por la situación de violencia modifica la trayectoria de vida y desarticula la identidad de las personas mayores. La pérdida de la casa o del hogar, significa algo más que perder la vivienda o el lugar de residencia, también el entorno doméstico, el entramado social en el que habitaban, los afectos, las costumbres, la geografía y, sobre todo, la desaparición de su lugar en el mundo ^29^. 

Las personas desplazadas se enfrentan a ambientes desconocidos, deben asumir nuevas actitudes y actividades en condiciones emocionales difíciles marcadas por perdidas e incertidumbres. Sin embargo, esta situación no es exclusiva de los adultos mayores, Bello ^30^ en un estudio con familias desplazadas menciona que ser desplazado significa haber perdido su lugar en el mundo, dejar de ser y estar en el lugar en el que se ha sido, es sinónimo de desarraigo, anonimato, dolor, rabia, presencia obstinada del recuerdo y esfuerzo de olvido. Por su parte Ocampo *et al*. ^5^, muestran que los contenidos acumulados en la memoria histórica como grupo o comunidad, que daban sentido a la existencia y aportaban elementos cognitivos y afectivos para definir un lugar para sí mismos en el mundo, se trastocan como referentes de orientación de la vida cotidiana. En este contexto, las personas que son víctimas de desplazamiento forzado experimentan la pérdida de elementos fundamentales como sujetos individuales y sociales, se ven obligadas a reelaborar cada vez su identidad y reafirmar lo que son en lo profundo de sí mismas, con su memoria y sus ilusiones, más allá de la supervivencia inmediata. 

Aunque campesinos e indígenas son desplazados de áreas rurales a urbanas, el impacto de este fenómeno no es el mismo. En Colombia, la definición de ruralidad ha sido, de manera típica, el residuo o complemento de la definición oficial de urbanización o cabecera ^31^. El censo discrimina positivamente en algunos casos a la población afro e indígena, pero no al campesinado. Suele decirse que la categoría “campesino” es ambigua, Parte del problema es que las estadísticas no reconocen en forma adecuada ni a los actores ni a los fenómenos políticos, mostrando una vez más la no valoración del campesinado por parte del Estado ^32^, no ocurre lo mismo con la poblacion indigena. 

Uno de los grupos que ha sido afectado especialmente por el desplazamiento forzado y la expropiación de sus territorios ancestrales son los pueblos indígenas y afro descendientes, situándose algunos de ellos al borde de la extinción ^33^. El territorio es parte del universo indígena y el indígena es parte integral del territorio; su proceso es indivisible. No se concibe el territorio sin los indígenas, y un indígena obligatoriamente está ligado a su territorio; esta consideración aferra el concepto diferenciador entre indígena y campesino, marca el uso del territorio como eje fundamental de supervivencia, de su vida; y no de su modelo o estilo de vida. El territorio figura un origen ancestral divino, localizado históricamente e identificado desde sus propios relatos de origen, habitación, uso y existencia. El territorio desde su concepción nunca será objeto de una negociación económica sobre su área o parte de su área, no se venderá y nunca se considerará como territorio a un espacio comprado. De él se desprenden una amplia gama de conocimientos y valores diarios que le dan sentido a su estructura cultural ^34^. Las montañas, los ríos, los desiertos, la selva, no son simples accidentes geográficos, sino recursos a los que históricamente se les han atribuido significados y funciones de regulación y protección. Su destrucción física, así como su uso inadecuado representan para muchas comunidades indígenas y campesinas la devastación de sus sistemas de conocimiento y protección.

De otro lado, en América Latina existen más de 800 pueblos indígenas que tienen una enorme diversidad territorial, demográfica y sociocultural, pero también tienen un denominador común: la exclusión y la pobreza material que los afecta con mayor intensidad que al resto de la población ^35^. Para gran parte de los indígenas, la verdadera vejez comienza cuando ya no se pueden realizar tareas o actividades para la mantención de la familia o para la reproducción material de la comunidad. El estatus y el rol social pueden aumentar a medida que se “envejece”, ya que las de más edad son las personas que atesoran la sabiduría y la memoria colectiva que debe transmitirse a los jóvenes para asegurar la reproducción cultural del grupo o pueblo. Por tanto, no cabe una interpretación “negativa” de la vejez, sino de continuidad cultural. Asimismo, muchas de estas personas son el vínculo entre las autoridades locales y la comunidad. Sumado a ello, debido a la migración de jóvenes y adultos, las personas de edad son las que se quedan en el campo y sostienen los proyectos de vida familiar por la vía de las estrategias económicas ^35^^,^^36^. Por consiguiente, la separación, destrucción o transformación del territorio de una comunidad indígena, es el mayor y principal determinante al generar rupturas a nivel social, económico, organizativo y psicológico. La fractura de esos sentidos y valores simbólicos a consecuencia del desplazamiento tienen un impacto mayor que en otros grupos poblacionales y las estrategias de adaptación de estas minorías a la vida urbana son más restringidas. Por ejemplo, Las formas occidentales de valorar con dinero los procesos, materiales, objetos y trabajos tienen una aplicación parcial en sus vidas. El dinero tiene valor solo en los procesos en los que se relacionan con su entorno social no indígena, y generalmente en los centros poblados. Ellos conjugan las formas primitivas de intercambios de materiales por materiales, trabajo por trabajo o cualquier de estas combinaciones que aplique según la necesidad de cada uno y no de la oferta. Ante la situacion de desplazamiento todo este referente se fractura. La situación de vulnerabilidad de las personas mayores indígenas se acrecienta en las ciudades ^37^^-^^39^. 

Cuando los mayores indígenas entran en las dinámicas de la ciudad, el sentido de “vejez” que se ha venido configurando desde los sustratos culturales de cada pueblo y la experiencia biográfica, debe empezar a re-acomodarse simbólicamente con la estructura social de la sociedad mayoritaria, es en ese punto, donde para la mayoría de pueblos indígenas se produce la ruptura y dislocación principal sobre el sentido de la vejez y el rol de ésta dentro de la sociedad ^36^.

Si bien el desplazamiento es principalmente una estrategia de protección y medios de vida, ser desplazado no es garantía de estar seguro o de lograr un medio de vida sostenible ^1^. Estudios en Colombia y Nepal encontraron que la propiedad de la tierra aumenta la probabilidad de desplazamiento, lo que sugiere que las personas que poseían la tierra podían esperar mayores amenazas por parte de actores armados, y que el temor de ser atacados superaba el riesgo de pérdida de tierra ^40^^,^^41^. Se ha demostrado que los ingresos y los privilegios económicos se asocian negativamente con el desplazamiento tanto en Colombia como en Nepal, lo que sugiere que los ingresos más bajos también implican una menor capacidad para adoptar medidas defensivas (por ejemplo, pagos de protección) o para soportar amenazas a sus medios de vida ^40^^,^^41^, aspecto crucial a la hora de salir del campo a iniciar una nueva vida en un entorno urbano; las condiciones de pobreza y marginación de los adultos mayores desplazados se incrementan exponencialmente.

Es evidente que los lugares a los que llegan los desplazados se encuentran habitados por las más heterogéneas comunidades, que incluyen además elementos pluriétnicos y multirregionales, cuyo referente común es la pobreza, históricamente experimentada en la urbe por personas y comunidades, pero desconocida para los recién llegados, procedentes en su mayoría del ámbito rural, donde cotidianamente encontraban satisfechas sus necesidades básicas. Las diversas fuentes de alimento que proporcionaba el campo y los amplios espacios geográficos, contrastan con los entornos urbanos de convivencia actual, lugares donde son frecuentes el hacinamiento y las dificultades para asegurar la alimentación cotidiana ^1^^,^^5^^,^^7^. 

No todas las personas desplazadas pueden lograr buenos niveles de integración económica, e incluso para quienes lo son, la integración económica no necesariamente equivale al bienestar económico ^1^. Los análisis estadísticos basados ​​en datos de Bosnia, Colombia y Sri Lanka sugieren que el hecho de ser un desplazamiento tiene un efecto negativo significativo en el bienestar económico ^41^^,^^42^^,^^43^. 

En lo que respecta al miedo, en casi todos los lugares donde el Grupo de Memoria Histórica ^10^ adelantó su trabajo, las víctimas refirieron el miedo como la emoción más constante y generalizada. Las víctimas, aun muchos años después de los hechos, expresaron que a pesar del paso del tiempo el miedo sigue presente en sus vidas. El miedo, mecanismo defensivo eficaz, se convierte en una emoción paralizante y mortificadora que impide que algunas personas puedan adelantar actividades esenciales para desarrollar sus vidas, como salir de sus hogares, caminar por el campo, reunirse con sus amistades. Lira y Castillo ^44^ hablan del miedo crónico, concepto en sí mismo contradictorio, ya que el miedo, como la angustia, son respuestas específicas ante una amenaza interna o externa percibida por el sujeto. Para las autoras, hablar de miedo crónico implica que éste deja de ser una reacción específica a situaciones concretas y se transforma prácticamente en un estado permanente en la vida cotidiana para cualquiera que se perciba amenazado. En el caso de los desplazados por conflicto armado, se demuestra que hay una continuidad del miedo que marca la experiencia del desplazamientos. El miedo, una emoción que se experimenta individualmente, se construye socialmente y se comparte culturalmente ^45^, resulta de las experiencias directas con el terror, las amenazas y la muerte antes de tener que huir de sus hogares. Esta emoción se acompaña de sentimientos de inseguridad y ansiedad, asociados con los trayectos de desplazamiento, la jornada del exilio y con los retos e incertidumbres de la llegada a un ambiente desconocido o, de la posibilidad de un redesplazamiento forzado ^46^. De acuerdo con James ^47^, el miedo organiza los afectos, lo que en un contexto del desplazamiento y cambio de las nociones de angustia, incertidumbre, seguridad y esperanza, le da expresión colectiva a estas experiencias. 

Aunque todos los participantes en este estudio hablan del lugar de origen con tristeza y añoranza y desean el retorno, no lo ven como una opción real. Arias et al, en un estudio sobre el deseo de retorno de personas desplazadas en Colombia, encontró que la tenencia de la tierra en el lugar de origen proporciona un incentivo para el retorno; los hogares vulnerables, en particular los encabezados por mujeres y los de minorías étnicas, buscan establecerse en el lugar de recepción y muestran un menor deseo de regresar; los desplazados como consecuencia de un ataque directo están menos dispuestos a regresar; las oportunidades económicas en el lugar de origen fomentan el retorno, mientras que las oportunidades económicas en el sitio de recepción disminuyen la disposición a regresar; y las redes sociales, como lo ejemplifica la membresía en organizaciones campesinas y la propiedad colectiva de la tierra, aumentan el deseo de regresar ^7^. Además, la decisión de regresar al lugar de origen está, por tanto, parcialmente determinada por las causas que llevan al desplazamiento y por el proceso mismo de desplazamiento. Esta selectividad implica que se deben tener en cuenta varios factores al evaluar los determinantes del deseo de retorno de la población desplazada, los adultos mayores participantes en este estudio pertenecen a minorías y fueron objeto de ataques directos, lo cual incrementa la vulnerabilidad y el miedo, y hace que, aunque se encuentren en condiciones muy desfavorables, el retorno al lugar de origen no sea visto como un opción viable. Para quienes han sido víctimas del desplazamiento forzado, el universo subjetivo da cuenta de un lugar al que no se quiere regresar, pues es sinónimo de dolor, miedo, inseguridad y peligro al haber dejado profundas heridas. El territorio físico, la montaña, el camino, se convirtieron en el lugar de la guerra. El patrimonio natural, los ríos, la vegetación, que daba un significado de libertad al lugar, al entorno en el cual se desarrollaba su vida, se convirtió en escenario de miedo y de terror.

La expulsión repentina los adultos mayores del propio territorio, como se ha mostrado en esta investigación, provee evidencias respecto a la situación que han vivido y resistido los pobladores rurales y los indígenas, como participantes, miembros y actores de la sociedad; como víctimas de una pérdida de poder y control sobre sus vidas en el torbellino de la violencia armada. 

Según Lechner ^48^ la interpretación del pasado a través de las experiencias condiciona las trayectorias futuras, dado que existe una relación entre tiempo, espacio y memoria. Los temores al futuro nacen en el pasado. Sin embargo, en la literatura acerca del desplazamiento no se han encontrado estudios ni referencias sobre cómo lidiar con el pasado ^49^. 

El adulto mayor desplazado envuelto en sus recuerdos histórico-culturales carga con su nostalgia por lo que le fue arrancado, único patrimonio con que cuenta para hacer frente a la condición de fragilidad física, económica y emocional; lleva consigo una serie de saberes y estrategias que le resultaban claves en su vida previa, que marcaban definitivamente la manera de ser, habitar y construir un espacio y un lugar en el mundo. Los barrios a donde llegan se convierten en espacio socio relacional donde convergen duelos, miedo, angustias, esperanzas, proyectos de vida, identidades regionales diversas; sus voces se deben seguir oyendo. 

La memoria, según ellos, debía hacerse en medio de la guerra, para detenerla, denunciarla, reclamar, transformar y construir la paz. Los adultos mayores son quienes, por lo general, resienten con mayor intensidad la salida forzada, sufren la experiencia como un profundo desarraigo, pues cuentan con pocos recursos físicos o cognitivos que posibiliten la adaptación. El deterioro de la calidad de vida, los cambios de clima, de alimentación y de hábitos acrecientan la sensación de vulnerabilidad e inestabilidad. Sin coordenadas ciertas y conocidas, las personas quedan a la deriva. Ni los paisajes, ni las costumbres, ni los sonidos, ni los colores, ni los olores, les resultan familiares. Todo ahonda la sensación de extrañamiento. El dilema entre la memoria y el olvido en una situación política traumática tiene connotaciones específicas. Recordar lo traumático puede ser imposible. Pero olvidarlo también puede serlo y el recuerdo puede volver violentamente a la memoria irrumpiendo sin tregua una y otra vez. 

Estas especificidades apuntan a la necesidad de que la programación de las intervenciones se diseñe con base en un análisis de las condiciones particulares, en lugar de herramientas, y con una contribución significativa de las propias personas desplazadas. El análisis del contexto en cualquier entorno dado debe proporcionar información para enfatizar soluciones duraderas.

Este estudio tienen varias fortalezas, da cuenta de la mirada cuidadosa y crítica con la que es necesario acercarse a la complejidad del desplazamiento forzado en Colombia y la vulnerabilidad de una minoría olvidada: adultos mayores campesinos e indígenas. Es un estudio cualitativo cuidadosamente diseñado y ejecutado que narra las vivencias y experiencias del desplazamiento forzado en una población que casi nunca se tiene en cuenta, a pesar de su triple vulnerabilidad: adulto mayor, desplazado y pobre. Aunque el estudio ha sido solo un acercamiento y sería importante reconocer y reconstruir con nuevos grupos de adulto mayores, su experiencia y su proceso, para de este modo tener un panorama abierto que permita tomar decisiones de política pública y generar recomendaciones pertinentes. Como lo menciona un habitante de Trujillo, Valle del Cauca, al grupo de memoria histórica: “… Si no se habla, si no se escribe y no se cuenta, se olvida y poco a poco se va tapando bajo el miedo …” ^10^

## Conclusión

Cuando hay desplazamiento, las personas mayores no solo se despojan de su tierra, de su casa, sino de su cosmos, de sus referentes vitales y se modifica su trayectoria de vida y su lugar en el mundo. Las intervenciones deben ser diseñadas basadas en análisis particulares y contextuales específicos.
